# Experiences of nurse educators regarding the use of the clinical skills laboratory at the School of Nursing in the Free State province

**DOI:** 10.4102/hsag.v28i0.2077

**Published:** 2023-02-13

**Authors:** Siphiwe T. Madlala, Agnes N. Mvandaba

**Affiliations:** 1 Department of Nursing Science, Faculty of Science, Agriculture and Engineering, University of Zululand, KwaDlangezwa, South Africa; 2 Department of Nursing Science, Free State School of Nursing, Eastern Campus, Phuthaditjhaba, South Africa

**Keywords:** clinical skills, clinical laboratory, nurse educator, student nurse, clinical practice

## Abstract

**Background:**

Integration of theory to practice by student nurses is a challenge in most training institutions accredited by the South African Nursing Council (SANC). Nurse educators require a fully equipped and functional clinical skills laboratory to impart clinical competency knowledge to student nurses.

**Aim:**

The purpose of this study was to understand the experiences of the nurse educators in teaching clinical skills to student nurses using the clinical skills laboratories.

**Setting:**

The study was conducted at the School of Nursing in the Free State province in 2021.

**Methods:**

A qualitative descriptive design was employed. Purposive sampling was used to select participants for the study. Unstructured one-on-one interviews were conducted with 17 nurse educators until data saturation was reached. Data were analysed thematically.

**Results:**

The three major themes that emerged during data analysis and were discussed to make recommendations of the study are as follows: clinical skills laboratory environment; human and material resources; financial constraints.

**Conclusion:**

This study revealed that there is a need for the use of the clinical skills laboratory by nurse educators to teach clinical practice to student nurses. Therefore, it is imperative that the study recommendations be considered for implementation to improve the use of the clinical skills laboratory.

**Contribution:**

The importance of integrating theory to practice by using the clinical skills laboratory during clinical practice teaching by nurse educators will be understood.

## Background

In South Africa, the nurse training institutions are undergoing a transformation to integrate nursing education with the Ministry of Higher Education and Training as part of a unified higher education system. This move required extensive changes in the training of nurses, which include re-curriculating the existing programmes to meet the higher education institutions’ (HEIs’) requirements. Over the past years, most nursing education institutions (NEIs), including the Free State School of Nursing, have been offering a comprehensive Diploma in Nursing (General, Community, Psychiatry) and Midwifery, which is a 4-year course as accredited by the South African Nursing Council (SANC) under Regulation R.425 of 22 February 1985, as amended (SANC 1985). In the year 2021, the Free State School of Nursing introduced a Diploma in Nursing: General Nurse at National Qualifications Framework (NQF) Level 6 offered under Regulation R.171 of March 2013.

The Diploma in Nursing (R.171 of March 2013) programme requires the integration of theory to practice and that the students be placed in accredited clinical areas for clinical practice, while clinical teaching using the clinical skills laboratory to integrate theory to practice was an option to orientate in these skills. Imran, Khan and Aftab (2016) agree that it is important for students to acquire clinical skills in the skills laboratory, thus making students acquire clinical skills before being exposed to real-life settings. The latter can only be achieved by incorporation of various teaching and learning strategies, including clinical placement of student nurses in clinical areas and the use of clinical skills laboratories.

The nurse training has been an on-the-job practice in the hospitals, but with the growing numbers of students enrolling in the nursing programmes recently, placement of students in clinical areas has become a challenge because of the limited number of available hospitals to accommodate all the students. Therefore, previously, nurse educators have been using the clinical skills laboratory as an additional clinical training environment. Currently, with the advancement of technology across the world, this has had a positive impact on the training of nurses using the clinical skills laboratory (Papastravrou et al. 2016).

Nationwide, nursing colleges are currently upgrading their clinical skills laboratories in preparation for accreditation by the SANC and movement to higher education for the training of new nursing qualifications, as proposed by the SANC to meet the required infrastructure for effective training of student nurses (Circular No. 1/2018). The design of a clinical skills laboratory should resemble a hospital ward as an accreditation requirement to optimise the simulation of clinical learning. It should be equipped with models and equipment like that used in patient care in the hospital units to ensure relevancy in clinical teaching and learning (Haraldseid, Friberg & Aase 2015). Furthermore, the clinical skills laboratory allows students to practise psychomotor and affective skills, acquire critical thinking skills, make mistakes, correct themselves and learn all the necessary steps required without the fear of posing life-threatening events to the patient (Papastravrou et al. 2016).

The nurse educators use clinical skills laboratories for demonstrating, practising and assessing students’ competencies in performing required procedures in various nursing disciplines. The simulation of skills by nurse educators to student nurses in a clinical skills laboratory helps to reduce anxiety in students prior to their physical contact with the patients (Van Vuuren 2016). Moreover, the use of the clinical skill laboratory by nurse educators is regarded as a suitable environment for teaching nursing students how to deal with real-life situations (Alnasir & Jaradat 2013). Teaching and learning experienced in the clinical skills laboratory form an important component of nursing education, considering that nursing is a practice-based profession that requires competent practitioners to render safe and quality nursing care to patients in clinical areas (Khoza 2015).

A clinical skills laboratory is regarded as a safe and controlled learning space that offers student nurses the opportunity to learn psychomotor skills and integrate theory with practice, allowing them to experience self-learning, and helps them to enhance their readiness for an actual clinical placement (Uysal 2016). Therefore, to achieve this, the use of the clinical skills laboratory requires competent nurse educators who are knowledgeable in using various teaching methods within such environments.

Although there are various challenges associated with the use of the clinical skills laboratory, which may hinder its effective use by nurse educators in teaching clinical practice to student nurses. These challenges include but are not limited to time constraints on using the clinical skills laboratory with limited resources, which is a challenging factor interfering with students’ acquisition of clinical skills (Haraldseid et al. 2015). Other challenges include a lack of funds, inadequate laboratory infrastructure, resistance to use the skills laboratory and poor technological literacy among the nurse educators (Elbireer et al. 2011).

Although nurse educators are exploring innovative ways to transition students successfully to clinical practice, there are limited studies conducted on experiences of nurse educators’ effective teaching in clinical skills laboratories (Petrova-Staykova, Von Stuward & Staykav 2017). The existing studies often targeted students’ perceptions of influencing factors, such as positive attitude, time, affiliation to the ward and personalisation of learning experiences (Haraldseid et al. 2015). This is confirmed by the study conducted in India, which revealed that student nurses perceived the use of simulation-based training in the skills laboratory as favourable in achieving good results on completion of their course (Raney et al. 2019). Little is known about the perceptions of nurse educators regarding the use of the clinical skills laboratory in teaching clinical practice to student nurses. Hence, this study focused on nurse educators’ experiences regarding the use of the clinical skills laboratory in teaching and learning of student nurses in the School of Nursing in the Free State province.

## Problem statement

Worldwide, nursing education consists of theory and practice components and covers cognitive, affective and psychomotor learning fields (Bayram & Caliskan 2020). Those learning fields cover the use of various teaching and learning strategies in different teaching environments, including clinical skills laboratories. The use of clinical skills laboratories requires competent nurse educators who are knowledgeable in using various teaching methods in different environments.

Most nurse educators are faced with challenges in teaching student nurses in a different environment, which is not a real hospital with real-life patients. Petrova Staykova et al. (2017) agree to the notion by stating that a different environment such as a clinical skills laboratory may negatively affect the nurse educators’ utilisation of this environment for teaching and learning of student nurses. Therefore, there are still gaps in the body of knowledge specifically focusing on nurse educators’ experiences regarding the phenomenon. Hence, the study focused on nurse educators’ experiences regarding the use of clinical skills laboratories in teaching and learning of student nurses.

## Purpose of the study

The purpose of the study was to explore and describe experiences of nurse educators in using the clinical skills laboratory during the teaching of student nurses.

### Setting

The study was conducted at the Free State School of Nursing, which consists of three subcampuses situated in the south, north and east of the Free State province. There are three clinical skills laboratories with about 270 student nurses and about 45 nurse educators at the school. The School of Nursing in the Free State province is accredited by the SANC for the training of student nurses under Regulation R.425 for a 4-year course leading to registration as a nurse (general, psychiatric or community) and midwife, or under Regulation R.683 for a 2-year course leading to registration as a general nurse (Bridging course), which are currently on a teach-out plan. Furthermore, the school offers a Diploma in Nursing, R.171 of March 2013, and Advanced Diploma in Midwifery, R.254 of 14 February 1975.

## Research design and methods

A qualitative descriptive design was used to examine the nurse educators’ experiences regarding the use of the clinical skills laboratory in teaching student nurses. According to Brink, Van der Walt and Van Rensburg (2017), a qualitative design can be used by the researcher to focus on the experience and understanding of participants’ perceptions on their actions. A descriptive design can find new meaning, describe what exists, establish how frequently the phenomenon occurs and classify the information (Burns & Grove 2011).

### Population and sample

The target population for this study was all the nurse educators employed at the School of Nursing in the Free State province with common experiences related to the phenomenon. The study participants were purposefully selected based on their rich knowledge and experiences regarding the use of the clinical skills laboratory.

#### Inclusion criteria

All nurse educators with above 2 years’ teaching experience, teaching any nursing subjects using the clinical skills laboratory, who consented to participate in the study and were working at the public School of Nursing in the Free State province were included in the study.

#### Exclusion criteria

All nurse educators who had never used the clinical skills laboratory for teaching student nurses, who had less than 2 years of teaching experience, who did not teach any nursing subjects, who were not willing to participate in the study and who were not working at the public School of Nursing in the Free State province were excluded from the study.

### Data collection

Unstructured individual face-to-face interviews were conducted with participants to collect data. Brink et al. (2017) indicate that during an unstructured interview, a specific question is posed by a researcher, followed by subsequent probing questions. The main question in this study was, ‘Tell me more about your experiences regarding the use of clinical skills laboratory in teaching of student nurses in the School of Nursing.’ This was followed by probing questions such as: ‘What challenges are you faced with in teaching student nurses using the clinical skills laboratory? What are your proposed suggestions to overcome these challenges to get more information from the participants regarding the phenomenon?’ An information letter explaining the purpose of the study was given to the participants willing to consent to participate in the study, followed by the consent forms.

The interviews were conducted in a private room in the participants’ offices during the time convenient to them. The interviews lasted about 30–45 min, conducted between 01 October 2020 and 30 November 2020. Chairs and a desk were placed at 1.5 m to observe social distance, the office window was opened, the interviewer and the participants wore masks throughout the interview and hand sanitisers were available for use, including cleaning the chairs and the desk after each interview. Furthermore, the participants adhered to coronavirus disease 2019 (COVID-19) regulations. Field notes were taken, and a voice-recorder was used to capture all interview sessions. Data were collected from 15 participants, with an additional 2 participants to ensure data saturation.

### Data analysis

Data analysis is a process involving images and making sense of the text (Botma et al. 2010). Data analysis was performed concurrently with data collection, and data were analysed thematically (Schmidt & Brown 2009). Seven steps of thematic analysis were followed to analyse the data, which were as follows: transcription; reading and familiarisation; coding; searching for themes; reviewing themes; defining and naming themes; and finalising the analysis (Schmidt & Brown 2009). The researcher listened to the audio recording, reading through the text and taking initial notes, and generally looking through the data to get familiar with it. Sections of the text and notes were highlighted to ensure coding. This was followed by reviewing of the themes, which was performed by looking over the codes created, identifying patterns among them and coming up with themes. The next step was going back to the data set and comparing it against the themes created. Defining and naming the themes involved formulating exactly what was meant by each theme and figuring out how it helped in understanding the data, including coming up with a succinct and easily understandable name for each theme. Then, this was followed by the writing up of themes to finalise the data analysis.

### Measures to ensure trustworthiness

Trustworthiness was ensured by adhering to the measures outlined by the model of Lincoln and Guba (Lincoln & Guba 1985), which are credibility, dependability, confirmability and transferability. These measures confirm the value of the study and substantiate the researchers’ honesty, truthfulness and loyalty to the participants during the research process. Credibility was ensured by interviewing all the nurse educators and recording the interview sessions until data saturation was reached after the 15th participants’ interview, which was confirmed by an additional 2 participants’ interviews to confirm data saturation. All transcribed data were further analysed by the author to verify and ascertain data codes, which was followed by a meeting with the researcher for discussion and reaching agreement regarding the codes to ensure confirmability. Dependability was ensured by the writing of thick descriptions of field notes and the use of a voice recorder to capture all interview sessions. The research methods were described to establish transferability.

### Ethical considerations

Ethical clearance was granted by the Research Ethics Committee of the University of Zululand (ref. no. 171110-030 PGM 2019/127); this step was followed by a request for permission to conduct the study from the Free State Department of Health Research Unit and the School of Nursing in the Free State. The participants were given information letters explaining the purpose of the study, and those who were interested to participate in this study signed the informed consent voluntarily, without being coerced. Their rights to autonomy and justice were upheld throughout the study by allowing them to participate voluntarily without risking prejudicial treatment and by treating them with respect and dignity (Polit & Beck 2018). Confidentiality, anonymity and privacy were also adhered to by using codes such as P1 during data analysis process, and all transcripts (including audio-recordings) were kept in a computer, which will be password protected in a locked office for a period of 5 years.

## Findings

Seventeen (*n* = 17) participants were interviewed during data collection, and their demographics are presented in Table 1.

**TABLE 1:** Demographics of the participants.

Variables	Value
**Gender**	
Men	6
Women	11
**Ethnicity**	
Black	15
White	2
**Educational qualifications**	
Degree in Nursing Education	14
Diploma in Nursing Education	3
Age range	Between 37 and 61 years

**Total participants**	**17**

Three major themes and subthemes are discussed as they emerged from the study. The study findings are summarised in Table 2.

**TABLE 2:** Themes and Sub-themes.

Major themes	Sub-themes
1. Clinical skills laboratory environment	1.1 Setting of the clinical skills laboratory 1.2 Access to the clinical skills laboratory
2. Human resources	2.1 Employment of staff (clinical instructors and support staff) 2.2 Clinical expertise of nurse educators
3. Financial constraints	3.1 Maintenance of skills laboratory equipment 3.2 Purchasing of skills laboratory equipment

### Theme 1: Clinical skills laboratory environment

There is inadequate space in most of the clinical skills laboratories, which results in the environment not being user friendly for clinical teaching and learning of student nurses. The setting and access to the clinical skills laboratory emerged as a subtheme.

#### Sub-theme 1.1: Setting of the clinical skills laboratory

Most participants stated that the way the clinical skills laboratories were designed was not user friendly for nurse educators to perform demonstrations to multiple levels of student nurses studying different disciplines such as midwifery, general nursing science, psychiatry and community nursing at the same time. There are no soundproof walls dividing the skills laboratory into rooms. This was evident in the following excerpts from the participants:

‘[…*E*]h currently we are unable to teach different levels of students with different disciplines such as psychiatry and midwifery at the same time because the skills laboratory is only divided by partitions. This makes it difficult to work as we are forced to wait for each other or used other means not teaching in the clinical skills laboratory.’ (P1, male, 60 years)

‘Mam, to be honest with you I don’t use the clinical skills laboratory because it is not user-friendly the way it is set up, one needs to wait for another nurse educator to finish teaching before you can be able to use it as there are no walls separating the rooms.’ (P3, female, 44 years)

‘At our campus, our clinical skills laboratory has no wall between the beds, they are separated by curtains which makes it difficult for more than one nurse educator to teach clinical practice at the same time. We must wait for one another to use the clinical skills laboratory.’ (P17, female, 39 years)

#### Sub-theme 1.2: Access to the clinical skills laboratory

Access to the clinical skills laboratories was viewed as the reason nurse educators effectively use the clinical skills laboratory. This was because the clinical skills laboratories have no delegated personnel to open and close them, even after hours, for nurse educators to use the clinical skills laboratory or for students to practise clinical skills. This was what the participants had to say:

‘[…*A*]t our campus we do not have any support staff allocated to open and close the skills laboratory for educators and students’ use after hours, the skills laboratory is only opened by the nurse educator only when she/he wants to use it for clinical teaching of students during working hours, unfortunately afterhours is locked.’ (P4, female, 52 years)

‘The clinical skills laboratory is only opened by nurse educators during working hours, after hours it is closed and there is no support staff available.’ (P2, female, 48 years)

‘[…*E*]h my challenge is when I want to access the clinical skills laboratory to demonstrate certain procedures to student nurse and leave them to practise after hours, there is no one to lock the clinical skills laboratory after hours. Therefore, access to our clinical skills laboratory is restricted only to during working hours.’ (P16, female, 41 years)

### Theme 2: Human and material resources

Human resources are constraints hindering the effective use of the clinical skills laboratory by nurse educators to teach clinical practice to student nurses. The subthemes that emerged were employment of staff (clinical instructors and support staff) and clinical expertise of nurse educators.

#### Sub-theme 2.1: Employment of staff (clinical instructors and support staff)

Most of the participants raised a concern about the absence of posts for clinical instructors in the staff establishment of the school. Participants verbalised this in the following statements:

‘In all the three campuses, there are no vacant posts for clinical instructors that will be strictly teaching student clinical practice in the clinical skills laboratory. … Eh … this is a problem as there is very limited number of nurse educators employed here who are also expected to also teach clinical practice. They hardly use the clinical skills laboratories due to time constrains.’ (P6, male, 49 years)

‘Mam, the entire school does not create and advertise posts for clinical instructors who will be strictly teaching clinical practice to student nurses in the clinical skills laboratory. We are expected to teach clinical practice in the clinical skills laboratory, but we hardly use the clinical skills laboratory because we do not have time and we are overworked.’ (P7, female, 45 years)

‘If they can employ clinical instructors who will be teaching clinical practice, I believe that the use of clinical skills laboratory will improve drastically as all nurse educators are trying their level best to use the clinical skills laboratory, but it is not effectively used.’ (P15, female, 49 years)

Other participants verbalised that there was no support staff employed to maintain the clinical skills laboratory. The concerns were captured as follows:

‘Our campuses do not have support staff such as clinical laboratory technicians, clinical skills laboratory manager nor clinical laboratory administrators who works in the clinical skills laboratory to ensure smooth running of the laboratory to support the nurse educators in using clinical skills laboratory to teach student nurses.’ (P5, Female, 37 years)

‘Our campus does not have the support staff such as laboratory technician to support nurse educators in using the clinical skills laboratory for teaching clinical practice to student nurses. We must do everything to make sure that we are using the clinical skills laboratory.’ (P11, Male, 41 years)

‘Since I arrived in this campus, I do everything when I am using the clinical skills laboratory to teach procedures to my students. There is no support staff available to ease the load. That’s demotivating.’ (P9, Female, 47 years)

#### Sub-theme 2.2: Clinical expertise of nurse educators

The lack of clinical expertise among nurse educators in using various teaching aids to teach clinical skills to nursing students in the clinical skills laboratory was identified as one of the challenges faced by nurse educators. This was what they had to say:

‘When teaching clinical skills in the skills laboratory, one needs to be an expert in using the available manikins. Therefore, sometimes I become less confident in using these manikins because of lack of knowledge on how to use them correctly hence I prefer to use real life patients at the hospitals.’ (P3, female, 44 years)

‘… Mam most of the manikins available in the clinical skills laboratory were not demonstrated to us by the representatives when they were purchased. Therefore, most of us we don’t know how they work which contributes to lack of the use of clinical skills laboratory in teaching student nurses.’ (P1, male, 60 years)

‘[…*E*]h Mam, to be honest with you, I don’t have much knowledge on how to use some manikins especially the high-fidelity ones. No one has ever demonstrated them to me since I arrived here. Therefore, I am not that confident in using the clinical skills laboratory to demonstrate procedures to my students, I rather go to the hospitals.’ (P8, female, 46 years)

### Theme 3: Financial constraints

There were challenges caused by financial constraints, which affect nurse educators’ abilities to effectively use the clinical skills laboratory for teaching clinical practice to student nurses. Maintenance of the skills laboratory equipment and purchasing of skills laboratory equipment emerged as the subthemes.

#### Sub-theme 3.1: Maintenance of skills laboratory equipment

It emanated from the interviews with the participants that the equipment used to teach student nurses clinical practice at the clinical skills laboratory was not maintained according to the manufacturer’s recommendations. This was expressed as follows:

‘The manikins (High-fidelity) and other equipment in the clinical skills laboratory are out of service …. None of them are being serviced as they are supposed to. This is mainly because of no money to service them to keep them within its life span. Therefore, some of this equipment are no longer working which impacts negatively in using the clinical skills laboratory.’ (P13, male, 40 years)

‘The equipment in our clinical skills laboratory is not being serviced. Most of them have lost its life expectancy and are no longer functioning well. This makes it difficult to use the clinical skills laboratory.’ (P10, female, 61 years)

‘The manikins in the clinical skills laboratory are old, dilapidated and others are not functioning because of lack of being serviced …. so that is the reason I hardly use the clinical skills laboratory.’ (P14, female, 60 years)

#### Sub-theme 3.2: Purchasing of skills laboratory equipment

Some of the participants raised concerns about the purchasing of new equipment for use in the clinical skills laboratory, which was problematic because of unavailability of funds. This was evident in the following excerpts:

‘Eh … Mam, I have been teaching at this campus for 10 years, we have been using the same equipment in this clinical skills laboratory every year without getting new updated technological equipment.’ (P4, female, 52 years)

‘We are told that the school does not have money to purchase new up to date and functional equipment. This affects us negatively in using the clinical skills laboratory with old or no equipment at all.’ (P17, female, 39 years)

‘We have not received new updated equipment for a long time because of unavailability of funds to purchase new equipment for the clinical skills laboratory. We are using old manikins which are not functioning properly, which affects the use of clinical skills laboratory.’ (P10, female, 61 years)

## Discussion

### Theme 1: Clinical skills laboratory environment

#### Sub-theme 1.1: Setting of the clinical skills laboratory

The study participants were concerned about the clinical skills laboratories’ design, which was not user friendly to them during the clinical practice teaching of student nurses of different levels studying different disciplines. Their clinical skills laboratories are divided by curtains, and some are partitioned without a soundproof partition. This negatively impacted the nurse educators’ use of clinical skills laboratories during clinical teaching of student nurses. Haraldseid et al. (2015) agree with this notion by stating that a poorly designed clinical skills laboratory with no soundproof partitions negatively impacts the process of demonstrating procedures to many students at the same time. Therefore, it is important to have a well-designed clinical skills laboratory with soundproof walls. In addition, the clinical learning environment and efficient clinical facilitation are essential elements in clinical teaching and have a considerable impact on students’ learning (Hoffman & Daniels 2020).

#### Sub-theme 1.2: Access to the clinical skills laboratory

Access to the clinical skills laboratory at any time by the students and the nurse educators was also a challenging factor, because the clinical skills laboratories were only accessible during working hours. This contributed to minimal use of the clinical skills laboratory by nurse educators to demonstrate clinical skills and allow students to practise these skills, even after hours, as there were no support staff allocated to maintain the clinical skills laboratories after hours. The study conducted by Relloso et al. (2021) revealed that the respondents stressed that additional time for an open laboratory hour enhanced their nursing skills through practising at the time convenient to them with no restriction on laboratory access. Hence, it is imperative to have support staff allocated to work at flexible hours to open and close the clinical skills laboratory, even after hours.

### Theme 2: Human and material resources

#### Sub-theme 2.1: Employment of staff (clinical instructors and support staff)

The findings of the study revealed that there were no clinical facilitators employed to teach clinical practice to student nurses in the clinical skills laboratory. The nurse educators were responsible for teaching theory and clinical practice to student nurses, which was viewed an excessive workload; hence, there was an inefficient use of the clinical skills laboratory. Moabi and Mtshali (2022) agree by stating that in order to ensure an effective use of the clinical skills laboratory, the NEIs need to increase the number of clinical instructors to mentor students. The clinical instructors are required to support students with developing their cognitive, psychomotor and affective skills, as well as offer emotional support during work-integrated learning as they perform clinical teaching at the patient’s bedside, as well as develop students’ skills in the clinical skills laboratory (Hoffman & Daniels 2020).

It became evident from the participants’ narratives that there is a shortage of staff who are clinical instructors and the support staff to assist nurse educators in teaching clinical skills to student nurses in the clinical skills laboratories. Similarly, the study conducted by Khoza (2015) agreed with the notion by stating that, indeed, the shortage of clinical instructors and laboratory technicians are major challenging factors leading to ineffective use of the clinical skills laboratory in teaching student nurses. The clinical environment is said to be effective when there are adequate material and human resources to support nurse educators to teach clinical learning activities to nursing students (Nachinab & Armstrong 2022). Therefore, employment of support staff is crucial to ensure support to nurse educators in effective use of the clinical laboratory as a teaching and learning environment.

#### Sub-theme 2.2: Clinical expertise of nurse educators

The lack of clinical expertise among the nurse educators in using the available high-fidelity manikins in the clinical skills laboratory was indicated as a barrier leading to ineffective use of clinical skills laboratories. It became evident from the participants’ responses that equipment such as high-fidelity manikins requires technological expertise, which was lacking among nurse educators. Mwale and Kalawa (2016) postulated that there is need for the nurse educators to be experienced, as experience facilitates acquisition of clinical skills that leads to the development of expertise. Therefore, a lack of nurse educators’ expertise in using high-fidelity manikins in teaching clinical practice to student nurses leads to loss of self-confidence and avoidance of using the clinical skills laboratory.

### Theme 3: Financial constraints

#### Sub-theme 3.1: Maintenance of skills laboratory equipment

The participants revealed that the equipment (high-fidelity manikins) used in the clinical skills laboratory was out of service, and some were no longer working properly because of a lack of services caused by unavailability of funds. This contributes to poor use of the clinical skills laboratory by nurse educators to teach student nurses clinical procedures. To provide quality clinical nursing training, nurse educators should have functional medical equipment (Relloso et al. 2021). Malfunctioning, old, obsolete and broken equipment becomes difficult to use to demonstrate critical clinical procedures to student nurses in the clinical skills laboratory (Moyimane, Matlala & Kekana 2017). Therefore, this posed major challenges to the effective use of the clinical skills laboratory to teach clinical practice to student nurses. Hence, it is important to adhere to the manufacturers’ routine servicing of high-fidelity manikins to keep their lifespan and improve their maximum performance.

#### Sub-theme 3.2: Purchasing of skills laboratory equipment

Purchasing of new equipment for the clinical skills laboratory was problematic at this school because of financial constraints. The participants highlighted that they have been using the same equipment for more than 10 years without getting new technologically updated equipment in their clinical skills laboratories; hence, there was a lack of interest in using the environment to teach student nurses. Medical equipment has a life cycle, requiring calibration, maintenance, repair, user training and finally retirement (Moyimane et al. 2017). Once they have reached the end of their lifespan, they need to be replaced with new advanced technological equipment. Therefore, using old, nonfunctioning equipment in teaching student nurses clinical practice skills in the clinical skills laboratory causes a barrier to use the clinical skills laboratory effectively. Moreover, it is imperative that the nursing school management budget for purchasing new technologically advanced equipment to be used in the clinical skills laboratory by nurse educators and that the nonfunctional equipment which is beyond repair should be discarded.

## Recommendations

It is recommended from this study that the clinical skills laboratory should be designed in a manner that is user friendly, with soundproof partitions for multiple teaching and learning of students in various levels when doing procedures for various disciplines, and it should be able to accommodate a sizeable number of students. The clinical skills laboratory should be flexible in terms of times to ensure that educators and student nurses can access the clinical skills laboratory at any time, including after hours, to teach, learn and practise clinical skills. Human resources should be increased by employing support staff to maintain the clinical skills laboratory and employ clinical instructors to teach clinical skills to student nurses to ease the workload from the nurse educators. New advanced technological manikins should be purchased, and the existing functional manikins should be regularly serviced to ensure that their life expectancy is kept up to date, and nonfunctional, dilapidated manikins should be discarded. Newly appointed nurse educators should be orientated or offered an in-service training on how to use the clinical skills laboratory, including the equipment, in teaching student nurses’ clinical practice. In addition, the school management should allow the necessary budget for the purchasing of new and technologically advanced manikins to replace the old and dilapidated manikins used by nurse educators in the clinical skills laboratory to teach student nurses.

## Conclusion

The use of the clinical skills laboratory by nurse educators to teach clinical practice to student nurses forms a crucial part of integration of theory to practice. The nurse educators at the School of Nursing in the Free State province are faced with challenges in using the clinical skills laboratories to teach student nurses. These challenges are multifactorial, including the design of the clinical skills laboratory, which is not user friendly for teaching clinical practice to various levels of students studying different disciplines at the same time because of a lack of soundproof walls. Access to the clinical skills laboratory is restricted to working hours, which is not accommodating of some nurse educators and students who may want to work after hours. Human and material resources require attention to ensure that nurse educators are using the clinicals skills laboratory optimally to teach clinical practice to student nurses. Moreover, there is a need to purchase new technologically advanced equipment and replace the old and nonfunctioning equipment in the clinical skills laboratory to promote quality teaching and learning by nurse educators in the clinical skills laboratory.
